# Is teledentistry effective to monitor the evolution of orthodontic treatment? A systematic review and meta-analysis

**DOI:** 10.1590/2177-6709.28.4.e2322195.oar

**Published:** 2023-09-15

**Authors:** Darlyane Kellen Barros TORRES, Milena Cristina Costa dos SANTOS, David NORMANDO

**Affiliations:** 1 Universidade Federal do Pará, School of Dentistry, Department of Orthodontics, (Belém/PA, Brazil).

**Keywords:** Teledentistry, Orthodontics, Systematic review

## Abstract

**Introduction:**

With the advent of COVID-19, teledentistry and remote monitoring have become an imminent reality that allows orthodontists to monitor orthodontic treatment through virtual checkups, which complement in-office appointments.

**Objective:**

To evaluate the effectiveness of using teledentistry in monitoring the evolution of orthodontic treatment.

**Material and Methods:**

Searches were performed in on-line databases. PECO strategy focused on comparing orthodontic patients exposed and not exposed to teledentistry. Searches and data extraction followed PRISMA guidelines. The assessment of the risk of bias and the certainty of the evidence was performed using the ROBINS-I and GRADE tools, respectively. A meta-analysis was also performed.

**Results:**

Out of 1,178 records found, 4 met the criteria and were included in the qualitative analysis. The risk of bias for follow-up assesment in aligner treatment was low to moderate; while for interceptive treatment, it was high. Studies are favorable to the use of teledentistry. The meta-analysis was performed with aligners studies only, due to heterogeneity. The certainty of the evidence was considered very low.

**Conclusion:**

With very low certainty of evidence, teledentistry using Dental Monitoring® software is effective as an aid in monitoring the evolution of interceptive orthodontic treatment (high risk of bias) and, especially, treatment performed with aligners (low to moderate risk of bias). The meta-analysis evidenced a reduction in the number of face-to-face appointments (mean difference = −2.75[−3.95, -1.55]; I^2^=41%; *p*<0.00001) and the time for starting refinement (mean difference = −1.21[−2.35, -0.08]; I^2^=49%; *p*=0.04). Additional randomized studies evaluating corrective orthodontic treatment with brackets and wires are welcome.

## RESUMO

**Introdução:**

Com o advento da COVID-19, a teleodontologia e o monitoramento remoto tornaram-se uma realidade iminente, permitindo ao ortodontista acompanhar o tratamento ortodôntico por meio de *checkups* virtuais, que complementam as consultas presenciais.

**Objetivo:**

Avaliar a eficácia do uso da teleodontologia no acompanhamento da evolução do tratamento ortodôntico.

**Material e Métodos:**

Buscas foram realizadas em bases de dados *on-line*. A estratégia PECO focou na comparação de pacientes ortodônticos expostos e não expostos à teleodontologia. As buscas e a extração dos dados seguiram a metodologia PRISMA. A avaliação do risco de viés e da certeza da evidência foi realizada com as ferramentas ROBINS-I e GRADE, respectivamente. Uma metanálise também foi realizada.

**Resultados:**

Dos 1.178 estudos encontrados, 4 atenderam aos critérios e foram incluídos na análise qualitativa. O risco de viés para a avaliação do acompanhamento de tratamentos com alinhadores foi baixo a moderado; enquanto para o tratamento interceptativo, foi alto. Os estudos são favoráveis ao uso da teleodontologia. A meta-análise foi realizada apenas com estudos de alinhadores, devido à heterogeneidade. A certeza da evidência foi considerada muito baixa.

**Conclusão:**

Com muito baixo nível de certeza da evidência, a teleodontologia utilizando o *software* Dental Monitoring® é eficaz para auxiliar no acompanhamento da evolução do tratamento ortodôntico interceptativo (alto risco de viés) e, principalmente, do tratamento realizado com alinhadores (risco de viés baixo a moderado). A metanálise evidenciou redução no número de atendimentos presenciais (diferença média = −2,75^ ^[−3,95, -1,55]; I^2^=^ ^41%; *p*<0,00001) e no tempo para início do refinamento (diferença média = −1,21^ ^[−2,35, -0,08]; I^2^=^ ^49%; *p*=0,04). Estudos randomizados adicionais avaliando o tratamento ortodôntico corretivo com braquetes e fios são bem-vindos.

## INTRODUCTION

Digital technologies have been used in all branches of Dentistry.^1^ In Orthodontics, teledentistry has become an imminent reality. Orthodontic treatment complemented by virtual checkups has been strengthened with the emergence of orthodontic aligners, due to remote monitoring technology being applicable for assessing the advance in treatment without the need for face-to-face appointments.^2^

Remote monitoring systems are part of Artificial Intelligence-Driven Remote Monitoring (AIRM).^3^ At its forefront, is the Dental Monitoring^®^ software (DM, Montreal, France), a software used on smartphones that allows the patient to accurately capture their teeth, in photos and videos, with the aid of a patented cheek retractor.^4^ However, studies demonstrate that, depending on the purpose of use, teledentistry can also be performed using photos and videos without the aid of a special retractor.^1^ ^,^ ^5^ ^-^ ^7^

Today there is a diversity of uses of teledentistry in orthodontics: Diagnosis, planning, consultation, monitoring of oral hygiene status, cooperation with elastics, evaluation of alignment or correction of malocclusion after the use of orthopedic appliances are some possibilities.^1^ ^,^ ^4^ ^,^ ^8^ ^,^ ^9^ The training of other professionals and remote assistance to a colleague are also situations in which this tool can also be incorporated.^10^

The challenges and limitations imposed by the COVID-19 pandemic have further stimulated the use of teledentistry.^7^ ^,^ ^11^ ^-^ ^12^ Remote checking, when possible, allows zero aerosol emission, reduced personal contact and zero risk of contagion.^7^ ^,^ ^12^ A recent study reported that 60% of American orthodontists have started using teledentistry, and 45% plan to keep it as part of their treatment routine.^13^ This opens perspectives for post-pandemic orthodontics with reduced number of face-to-face appointments, without impacting the quality of treatment.^14^

Systematic reviews^15^ prove that the use of teledentistry is effective, and can be comparable to in-person screening, especially in school programs, rural areas, and areas with limited access to health care. However, to date, no systematic reviews have been identified on the use of teledentistry focusing on the evolution of orthodontic treatment.

Therefore, the aim of the present systematic review is to evaluate, through controlled clinical studies (randomized or not) the effectiveness of teledentistry to monitor the evolution of orthodontic treatment.

## MATERIAL AND METHODS

This systematic review was reported in accordance with the PRISMA 2020 guidelines (Preferred Reporting Items for Systematic Review and Meta-Analysis, available at www.prisma-statement.org).^16^

### ELIGIBILITY CRITERIA

The eligibility criteria followed the format of the PECO strategy. Studies that met the following selection criteria were included:

1. Participants: patients undergoing orthodontic treatment, with brackets, orthopedic appliance or orthodontic aligners, and without restriction of sex, age, race and malocclusion. Syndromic patients were excluded.
2. Exposure: remote monitoring of the evolution of orthodontic treatment, using photos, videos or smartphone apps.
3. Comparison: group with treatment monitoring performed exclusively face-to-face or dentofacial measurements performed in a conventional way.
4. Outcome: effectiveness of teledentistry (via photos, videos or applications) to monitor the evolution of orthodontic treatment performed with brackets, orthopedic appliance or orthodontic aligners. Studies in which teledentistry was used for diagnosis and orthodontic treatment planning, or as a patient motivation tool, were excluded.
5. Study types: randomized clinical trials and controlled clinical trials. Case series, case reports, expert opinions and reviews were excluded.

### INFORMATION SOURCES, SEARCH STRATEGY AND STUDY SELECTION

Electronic searches were performed according to the PECO strategy, from September to November 2021 and checked again in July 2022. Five databases were searched: PubMed (Medline), Scopus, Web of Science, Cochrane and Virtual Health Library (Supplementary Tables 1 and 2). Other search methods were also performed: OpenGrey, Google Scholar, Clinical Trials, reference lists and alerts received until March 2023.

**Table 1:** Studies excluded after reading the articles in full, as they did not meet the eligibility criteria.

Study	Reason for exclusion
Hansa *et al*.^2^, 2018	According to the corresponding authors, this study presents the same sample of a more recent study included in this systematic review.
Berndt *et al*.^10^, 2008	Although teledentistry has been used with video-conferences, the main objective was to compare the treatment performed by orthodontic students and by general practitioners.
Morris *et al*.^19^, 2019	The study did not evaluate telemonitoring in orthodontic patients. The sample was with typodonts.
Putrino *et al*.^11^, 2020	These were not controlled clinical studies.
Saccomanno *et al*.^12^, 2020	
Impellizzeri *et al*.^20^, 2020	
Ackerman^21^, 2019	

**Table 2:** Data extraction from the studies included in this systematic review.

Author, year and type of study.	Sample (n) / age ± SD	Orthodontic treatment	Telemonitoring method	Comparison method	Evaluated variables / statistics	Results	Conclusion	Funding source	Conflict of interest report
Kuriakose *et al*.^22^ (2019) Case-control (prospective)	Patients in mixed or permanent dentition. Malocclusion: NR. Single group (n=20) / 11.5 (NR) years.	Hyrax^®^ expander with or without brackets bonded to upper and/or lower teeth, to correct crossbite or improve dental arch shape.	Dental monitoring® (DM).	Measurements in intraoral exams and in digital models of the same patients.	Intraclass correlation for intermolar width in the maxillary dental arch (mm). Correction of posterior crossbite. / linear association. Significance level: NR.	There was no significant difference between the modalities (p-value: NR).	Personal assessment of maxillary expansion with a Hyrax® expander can be replaced by remote monitoring using DM software, but challenges associated with digital image quality make it difficult to use this remote assessment for some patients.	None	None
Moylan *et al*.^23^ (2019) Case-control (prospective)	Patients in mixed or permanent dentition and in need of maxillary expansion. Malocclusion: NR. Single group (n=12) / 10-17 (NR) years.	Hyrax^®^ expander.	Dental monitoring^®^ (DM).	Measurements on plaster models of the same patients.	Bland-Altman agreement analysis for intercanine and intermolar widths in the maxillary dental arch. Significance level: NR.	Intercanine and intermolar measurement differences averaged 0.17 mm and -0.02 mm, respectively, and were considered non-significant. (p-value: NR).	As long as the quality of the video scans is acceptable, the use of monitoring software can be trusted for follow-up and clinical decision making.	VCU Alexander Fellowship, Southern Association of Orthodontists, and Virginia Orthodontic Education and Research Foundation.	NR
Hansa *et al*.^14^ (2020) Case-control (retrospective)	Patients in permanent dentition. Class I, II or III malocclusion. Telemonitored group (n=88) / 25.3 ± 11.1 years. Control group (n=67) / 25.4 ± 10.1 years.	Therapy with Invisalign orthodontic aligners for correction (up to first molars) without extractions and with 30 to 65 initial aligners.	Dental Monitoring^®^ (DM), GoLive^®^ option, specific for aligners.	Group with exclusively face-to-face monitoring.	Treatment duration (months), number of refinements, number of aligners for refinement, time to first refinement, and number of face-to-face visits. / Independent t and Mann-Whitney tests with a 5% significance level.	The DM group had 2.26 (23%) fewer visits, compared to the control (7.56 vs. 9.82; p<0.001). There were no significant differences between the DM and control groups, respectively, with regard to treatment duration (14.58 vs. 13.91), number of refinements (1.00 vs. 0.79), number of aligners refinement (19.91 vs. 19.85) and time to first refinement (9.46 vs. 9.97).	The DM group had a significantly reduced number of visits, compared to the control group, over the duration of treatment. There were no significant differences between the two groups in treatment duration, number of refinements, number of refinement aligners, or time to first refinement.	none	none
Hansa *et al*.^24^ (2021) Case-control (restrospective)	Patients in permanent dentition. Class I, II or III malocclusion. Telemonitored group (n=45) / 30.1 ± 13.7 years. Control group (n=45) / 31.0 ± 11.5 years.	Therapy with Invisalign orthodontic aligners, for correction (up to second molars) without extractions and with 15 to 50 initial aligners.	Dental Monitoring® (DM), with face-to-face consultations at 16-week intervals and aligners change every 7 days or as visualized by telemonitoring.	Group with exclusively face-to-face follow-up, with consultations at approximately 6 to 8 week intervals and aligners exchange performed every 7 days.	Treatment duration (months), number of refinements, number of aligners for refinement, time to first refinement, number of in-person visits, and differences between predicted and achieved tooth positions. / Independent t and Mann-Whitney tests, with a 5% significance level.	There was a significant reduction (p = 0.001) in the number of consultations in 3.5 (33.1%) in the DM group. There was also a significant reduction (p=0.001) in time to first refinement (1.7 months) in the DM group. Compared to the predicted tooth positions by Invisalign®, the actual tooth positions were statistically (p < 0.05) more accurate for the DM group for the maxillary anterior dentition in rotational movements and mandibular anterior dentition for labiolingual linear movement.	DM significantly reduced the number of office visits by approximately 3.5 visits (33.1%) over the course of treatment. The duration of the first refinement was (significantly) reduced by 1.7 months (28%) in the DM group. This achieved clinically similar accuracy between the 2 groups in less time, indicating improved tracking for the DM group. Treatment duration, number of refinements, number of refinement aligners, or number of emergency visits were similar between groups.	none	none

SD = standard deviation; DM = dental monitoring; NR = not reported.

Authors DT and MS independently classified the articles by title, abstract and full text, using the bibliographic reference manager Endnote (version X7, Thomson Reuters), according to the proposed topic and eligibility criteria. Disagreements during study selection were resolved through a consensus meeting and, when appropriate, through consultation with the third author (DN). No restrictions on date or language were applied.

### DATA ITEMS AND EXTRACTION

The following data were extracted from the included studies: author and year, sample and age, orthodontic treatment performed, telemonitoring method, comparison method, evaluated variables, results and conclusion. When necessary, an email was sent weekly (for three weeks) to the authors, to recover missing information. Two authors (DT and MS) tabulated the data extraction, individually. A comparison of all the information obtained was made. Disagreements during data extraction were resolved through a consensus meeting and, when appropriate, through consultation with the third author (DN).

### RISK OF BIAS ASSESSMENT

Randomized studies were not found, only controlled clinical trials. Therefore, the articles included were evaluated for risk of bias using the Cochrane ROBINS-I tool for non-randomized intervention studies.^17^ The evaluation was performed independently by two authors (DT and MS). By means of consensus meeting, a third author (DN) intervened for the final decision, in case of disagreements.

The Cochrane ROBINS-I tool has three main assessment domains: pre-intervention, during the intervention and post-intervention. After individualizing the main criteria, the risk of bias was assessed for each level of each domain, and classified as “low”, “moderate”, “serious”, “critical” or “no information”. Finally, an overall judgment of the risk of bias for each study was generated.

### METHODS OF SYNTHESIS AND ASSESSMENT OF CERTAINTY OF EVIDENCE

Data from the included studies were analyzed using the Review Manager software (Review Manager v. 5.3, The Cochrane Collaboration; Copenhagen, Denmark) to evaluate the common outcomes tested among the studies and related to exposure and non-exposure to teledentistry. The mean and standard deviation of the score of each test, and the total number of individuals in the control and DM groups were used. The mean difference (MD) was applied, with 95% confidence interval (95% CI). If some of the information needed for the meta-analysis was absent from the selected studies, the authors were contacted to provide the missing data. Heterogeneity was tested using the I^2^ index. The random effect model was used in all meta-analyses because the studies were not functionally equivalent, in this, objective to generalize the findings from the meta-analysis.

To assess the level of certainty of the evidence, the Grading of Recommendations, Assessment, Development and Evaluation Pro software (GRADEpro Guideline Development Tool, available online at https://gradepro.org/) was used.^18^ It classifies the quality of evidence into four levels: very low, low, moderate, and high. “High quality” suggests that the actual effect is close to the estimate of the effect; “Very low quality” suggests that there is very little confidence in the effect estimate, and the reported estimate may differ materially from that measure. This tool considers four aspects to classify the quality of evidence: “Certainty Assessment”, “Impact”, “Certainty” and “Importance”.

## RESULTS

### SELECTION OF STUDIES

**Figure 1:**
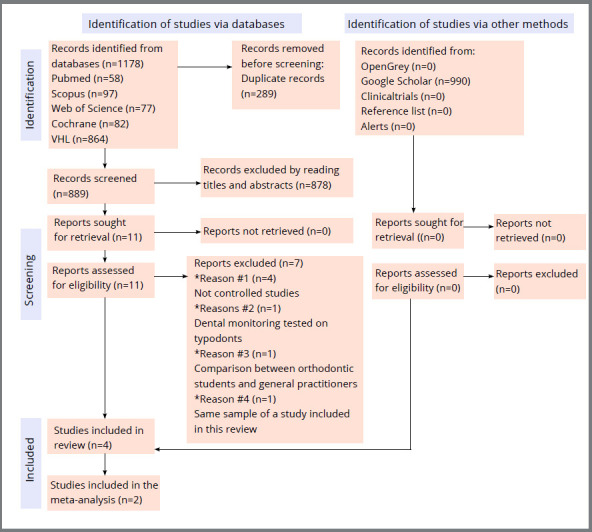
Flow diagram for the study selection procedure.

A total of 1,178 records were retrieved after the searches in the databases. After duplicate removal, 889 records were screened by reading the titles and abstracts. Eleven articles were assessed for eligibility, and seven were excluded (Table 1): one had the same sample of another included study (information obtained via contact with the corresponding author),^2^ one compared orthodontic students with general practitioners,^10^ one tested Dental Monitoring^®^ on typodonts,^19^ and four did not have a control group.^11^ ^,^ ^12^ ^,^ ^20^ ^,^ ^21^ An additional 990 documents were identified via other methods, but none were eligible after reading the titles and abstracts. Finally, four articles^14^ ^,^ ^22^ ^-^ ^24^ were selected, all from databases. The study selection process is presented in Figure 1. The summaries of the characteristics and results of the articles are presented in Table 2.

### CHARACTERISTICS OF THE STUDIES

All studies were non-randomized controlled clinical trials^14^ ^,^ ^22^ ^-^ ^24^ and used the same telemonitoring method: measurements performed with the aid of Dental Monitoring^®^ software. Two studies were prospective, in which the samples comprised patients in mixed or permanent dentition who were treated with maxillary expansion.^22^ ^,^ ^23^ There was divergence in the method of comparison: one study used measurements by intraoral exams and digital models,^22^ while the other used plaster models.^23^ The analysis was performed by intermolar^22^ ^,^ ^23^ and intercanine measurements in the maxillary dental arch.^23^

The other two studies included^14^ ^,^ ^24^ were from the same research group, being retrospective, and the samples comprised patients in permanent dentition treated with Invisalign orthodontic aligners. The corresponding author confirmed, by email in August 2021, that such studies had different samples. In the 2020^14^ study, the correction was extended to first molars; and in the 2021^24^ study, it also incorporated second molars. Treatment duration (months), number of refinements, number of aligners for refinement, time to first refinement, number of face-to-face visits and differences between predicted and achieved tooth positions were evaluated - this last item was evaluated only in the 2021 study.

### RISK OF BIAS IN INDIVIDUAL STUDIES

Table 3 describes the risk of bias (Rob) analysis of the four studies included in this systematic review, performed using the ROBINS-I tool. One study was classified as low risk,^24^ as it presented “low Rob” in all evaluated categories. One presented moderate risk,^14^ due to not specifying the proposed time of exchange between aligners and the time between face-to-face visits, presenting “moderate Rob” in only one category. The other two studies were classified as serious risk of bias,^22^ ^,^ ^23^ for presenting “moderate Rob” in at least three categories or at least one “serious Rob”. This classification was due to the sample size not being representative of the population,^22^ ^,^ ^23^ as well as the report of many losses,^22^ non-specification of the rapid maxillary expansion (RME) protocol,^22^ ^,^ ^23^ in addition to reports of a varied prescription of RME and the association of other types of treatment,^22^ no description of previous calibration by the examiner,^22^ and results without indication of *p*-values and significance level.^22^ ^,^ ^23^

**Table 3:** Quality assessment of the ROBINS-I tool for all non-randomized studies.

	**Kuriakose *et al*.** ^22^ **(2019)**	**Moylan *et al*.** ^23^ **(2019)**	**Hansa *et al*.** ^14^ **2020**	**Hansa *et al*.** ^24^ **2021**
PRE-INTERVENTION				
Bias due to confusion	Moderate Rob	Moderate Rob	Low Rob	Low Rob
Bias in the selection of participants for the study	Low Rob	Low Rob	Low Rob	Low Rob
IN THE INTERVENTION				
Bias in the classification of interventions	Moderate Rob	Moderate Rob	Moderate Rob	Low Rob
POST-INTERVENTION				
Bias due to deviations from the intended intervention	Serious Rob	Low Rob	Low Rob	Low Rob
Bias due to missing data	Low Rob	Low Rob	Low Rob	Low Rob
Bias in measuring results	Moderate Rob	Low Rob	Low Rob	Low Rob
Bias in the selection of the reported result	Moderate Rob	Moderate Rob	Low Rob	Low Rob
Overall Rob Judgment	Serious Rob	Serious Rob	Moderate Rob	Low Rob

Rob = risk of bias.

### INDIVIDUAL RESULTS OF STUDIES AND SYNTHESIS

Studies with maxillary expansion^22^ ^,^ ^23^ found that monitoring software seems to provide an accurate assessment of linear tooth movements,^23^ and that Dental Monitoring^®^ can remotely identify posterior crossbite correction.^22^ Regarding the two studies in which orthodontic treatment was performed with aligners, it was reported that, in both studies,^14^ ^,^ ^24^ the telemonitored group had fewer face-to-face consultations, compared to the control group. The 2020^14^ study reported a 1.26 (23%) reduction in visits, compared to the control (7.56 vs 9.82; *p*<0.001); and the 2021^24^ study reported a reduction in the number of visits by 3.5 (33.1%) in the DM group, compared to the control group (*p*=0.001). There were no differences between the DM and control groups, respectively, regarding treatment duration (14.58 vs 13.91), refinements (1.00 vs 0.79) and number of refinement aligners (19.91 vs 19.85).^14^ Both studies evaluated the time of first refinement, with divergent results. The first study^14^ did not notice significant differences between the groups (9.46 vs 9.97; *p*>0.05), and the second one^24^ reported a significant reduction (*p*=0.001) in the time to first refinement: 1.7 months shorter in the DM group. Furthermore, compared to the tooth positions predicted by Invisalign^®^, the actual tooth positions were statistically (*p*<0.05) more accurate for the DM group for the maxillary anterior dentition in rotational movements and mandibular anterior dentition for buccal-lingual linear movement.^24^

**Figure 2:**
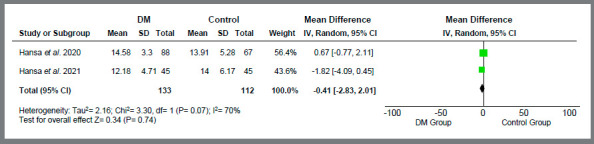
Forest plot of the difference in treatment time in months for the use of teledentistry between the “Dental Monitoring” and “Control” groups. 95% confidence interval and 95% prediction interval.

**Figure 3:**
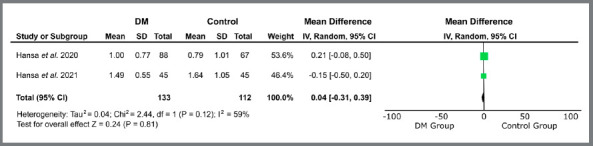
Forest plot of the difference in the number of refinements for the use of teledentistry between the “Dental Monitoring” and “Control” groups. 95% confidence interval and 95% prediction interval.

**Figure 4:**
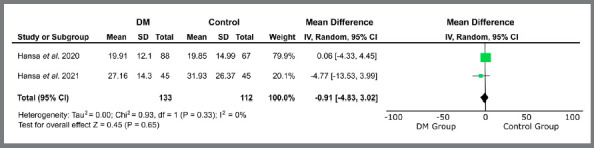
Forest plot of the difference in the number of refinement aligners for the use of teledentistry between the “Dental Monitoring” and “Control” groups. 95% confidence interval and 95% prediction interval.

**Figure 5:**
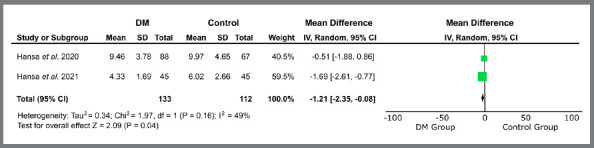
Forest plot of the difference in time for the first refinement regarding the use of teledentistry between the “Dental Monitoring” and “Control” groups. 95% confidence interval and 95% prediction interval.

**Figure 6:**
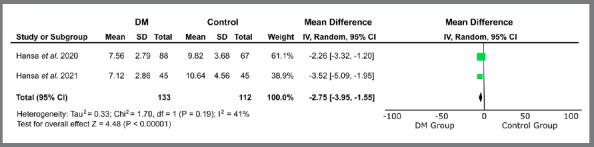
Forest plot of the difference in the number of face-to-face consultations regarding the use of teledentistry between the “Dental Monitoring” and “Control” groups. 95% confidence interval and 95% prediction interval.

Only studies related to treatment with orthodontic aligners proceeded to quantitative analysis, due to methodological similarity. The variables “treatment time” (MD = -0.41 [-2.83, 2.01]; I^2^= 70%; *p*= 0.74) (Fig 2), “number of refinements” (MD = 0.04 [-0.31, 0.39]; I^2^= 59%; *p*= 0.81) (Fig 3), “number of refinement aligners” (MD = -0.91 [-4.83, 3.02]; I^2^= 0%; *p*= 0.65) (Fig 4), “time for the first refinement” (MD = -1.21 [-2.35, -0.08]; I^2^= 49%; *p*= 0.04) (Fig 5), and “number of appointments” (MD = -2.75 [-3.95, -1.55]; I^2^= 41%; *p*<0.00001) (Fig 6) were investigated. The last two variables were statistically significant and favorable to the use of teledentistry via DM to monitor orthodontic treatment performed with aligners. Studies related to interceptive treatment were eliminated, due to heterogeneity in analysis data. Kuriakose *et al*.^22^ evaluated agreement via intraclass correlation and Moylan *et al*.,^23^ via Bland-Altman plot. Both did not assess the relationship between teledentistry and treatment time - the only variable that would be possible to measure together with the other two studies.

The certainty of the evidence was rated as low (Table 4). The risk of bias seriously affected the evidence, due to methodological limitations present in the studies, mainly those related to interceptive treatment. The consistency was seriously affected due to the heterogeneity regarding the type of orthodontic treatment performed, methodology and data analysis performed.

**Table 4:** Result of the GRADE assessment of certainty of evidence.

CERTAINTY ASSESSMENT				IMPACT			CERTAINTY	IMPORTANCE	
№ of studies	Study design	Risk of bias	Inconsistency	Indirect evidence	Imprecision	Other considerations			
4	Controlled clinical studies	serious^a^	serious^b^	not serious	not serious	none	The smartphone software Dental Monitoring^®^ can be used for measuring and monitoring maxillary expansion. (Kuriakose *et al*.^22^, 2019 and Moylan *et al*.^23^, 2019). There were no significant differences in treatment time between patients who received or did not receive telemonitoring, but the number of face-to-face consultations was considerably reduced (Hansa *et al*.^14^, 2020 and Hansa *et al*.^24^, 2021).	⨁◯◯◯ Very low	IMPORTANT

^a^ Two studies have a high risk of bias, one a moderate risk, and only one study was classified as a low risk of bias. The tool used was “ROBINS-I”.

^b^ Heterogeneity in the type of orthodontic treatment performed, methodology and data analysis.

## DISCUSSION

Technologies are becoming common in the orthodontic profession. Intraoral scanning, diagnosis with the aid of tomographic imaging, 3D-printing and aligner manufacturing are some examples - most of them requiring exclusive skill from the professional.^25^ Teledentistry, on the other hand, is able to welcome the patient, in partnership with the professional, as the protagonist of their own orthodontic treatment. The impossibility of face-to-face care during the beginning of the COVID-19 pandemic led orthodontists to offer virtual control of treatment to patients. During this period, some cross-sectional descriptive studies were carried out to evaluate the topic, and demonstrated that teledentistry is a viable solution in emergency situations, and can also be considered for normal times.^7^ ^,^ ^11^ ^,^ ^12^

A scoping review^26^ noted that teledentistry (with the aid of smartphones) in orthodontics was being used through cephalometric diagnostic apps (six studies: 35%), apps used as reminders (seven studies: 41%), and remote monitoring via apps (four studies: 24%). These last four studies reviewed Dental Monitoring^®^: one study^19^ is part of our list of excluded studies, for not meeting the eligibility criteria; and three studies^14^ ^,^ ^22^ ^,^ ^23^ were included in the present systematic review.

This systematic review aimed to analyze only randomized clinical studies or those with the presence of a comparison group related to Orthodontics, more specifically to the monitoring of treatment evolution. The included studies concluded that teledentistry is effective in monitoring orthodontic treatment. However, in all the situations found, it is possible to perceive a requirement for some type of previous training, either by the orthodontist or mainly by the patient. When records are made without experience, even with help of someone at home, reliability and accuracy can be questionable.^27^

The four studies^14^ ^,^ ^22^ ^-^ ^24^ used Dental Monitoring^®^ as a telemonitoring method. Although the models generated by photos and videos are accurate enough for clinical applications,^19^ the use of this tool should be taught to patients or guardians with certain training, so that, alone or with the help of family members, they can obtain adequate images and videos. It is essential that the orthodontist also be able to motivate patients, parents or guardians throughout the treatment, in order to avoid non-cooperation.

Among the studies included in this systematic review, only one study^22^ reported the reasons for refusing to participate in the research, such as: parents or guardians find it difficult to use the app or did not have a smartphone or did not feel confident with remote monitoring. This study also found dropouts due to inability to properly use the software or follow the proposed scanning protocol (33.3% of an initial sample of 30 participants). Orthodontists who intend to use teledentistry may face similar situations. However, research directly related to patient satisfaction in the use of teledentistry in times of COVID-19 demonstrated that most patients express positive opinions and ease of use.^28^ ^,^ ^29^ Both the orthodontist and the patient must be able to effectively use the chosen telemonitoring tool.

Regarding the types of orthodontic treatment evaluated in the studies, these were limited to interceptive^22^ ^,^ ^23^ or the use of orthodontic aligners.^14^ ^,^ ^24^ No studies were found with conventional corrective or compensatory mechanics (brackets and wires), except for the 4x2 alignment, which was of low complexity and was characterized as a type of interceptive treatment. Some articles showed^22^ ^,^ ^23^ that not only the most modern treatments, such as those performed with orthodontic aligners, are subject to the use of technology to monitor patients. Teledentistry can provide fewer visits to the clinical environment, which often generates fear, apprehension and discomfort, especially for children patients. 

Remote monitoring of rapid maxillary expansion proved to be effective^22^ ^,^ ^23^ not only for checking posterior crossbite correction, but also for evaluating linear measurements, compared to traditional methods, which may be important to quantify the gains achieved. Differences between methods appear not to be clinically important. One of these studies^22^ showed that 43% of patients preferred remote follow-up for the assessment of expansion than face-to-face, and 18% reported not having a preference between the two options.

It was also possible to notice that the constant remote monitoring can improve the cooperation of the patient who uses the orthodontic aligner. One of the studies^24^ observed that the group that used Dental Monitoring^®^ achieved greater precision in movements than the group monitored in person. Based on this, it is understood that the remote monitoring of the patient can bring more commitment and better use of aligners. This greater precision of movement was restricted to the region of maxillary and mandibular incisors, and this may have caused a reduction in the time for the beginning of refinement in the telemonitored group - a possible effect of a more intense monitoring and early detection of problems such as loss of tracking movement or lack of proper alignment of the aligner.^24^ The patient monitored at distance seems to be more committed to the treatment than the patient monitored only in person. This can also be observed in studies that evaluated the performance of oral hygiene and the formation of bacterial plaque.^30^ ^-^ ^32^

The lower number of visits among the groups remotely monitored also seems to be an interesting finding, as this can be an excellent option for patients who live in other cities or to assist those who cannot attend the monthly visit for some reason. This practice can bring benefits to the progress of treatment, especially knowing that orthodontic patients were satisfied with virtual consultations through videos and that they would prefer to have more consultations carried out remotely, being more convenient for them.^28^

Dental Monitoring^®^ was the telemonitoring method of choice for all studies included in the present review. The incorporation of this tool seems to have increased with the advent of aligners. In conventional orthodontics, it seems to be an excellent option for controlling side effects, detecting passive arches, monitoring tooth eruption and identifying bracket fractures^2^ ^,^ ^11^.

The use of artificial intelligence for remote monitoring gained notoriety, especially during the COVID-19 pandemic.^33^ ^,^ ^34^ The use of these technologies can incur additional costs for the orthodontist, but simple options, such as the patient sending photos and videos through e-mails^5^ or applications such as Whatsapp,^35^ ^,^ ^36^ can be a simple and low-cost solution. It is expected that technologies and apps that are normally part of our daily lives, due to their ease of use, can be used as an aid in monitoring any type of orthodontic treatment.

### LIMITATIONS

The restricted number of studies found, the methodological heterogeneity and the very low certainty of the evidence, limit the ability to generalize the present results, reducing the possibility of associating clinical significance regarding the effectiveness of teledentistry to monitor the evolution of orthodontic treatment in all the modalities.

### DIRECTIONS FOR FUTURE RESEARCH

Controlled or randomized studies with smartphone apps commonly used during fixed orthodontic treatment are welcome. As a suggestion for future studies, it would be interesting to evaluate the effectiveness of sagittal elastics with and without remote monitoring; also, the monitoring of tooth mobility during orthodontic forces application in patients with a history of periodontal disease. With the increasing demand for teleorthodontics, it is expected that new technologies associated with artificial intelligence will emerge and be part of future research.

## CONCLUSIONS

The studies included in the present review were controlled clinical trials with low to high risk of bias, and very low certainty of evidence. Teledentistry using the Dental Monitoring^®^ software is effective in helping to monitor the evolution of interceptive orthodontic treatment (high risk of bias), and especially in the treatment performed with orthodontic aligners (low to moderate risk of bias). The meta-analysis showed that teledentistry during orthodontic treatment with aligners reduces the time to start refinement and also the number of face-to-face visits, not being able to affect the total treatment duration, the number of refinements and the number of refinement aligners. Randomized studies evaluating usual technological alternatives among patients and orthodontists are welcome, especially in orthodontic treatment with fixed appliances, due to the lack of studies with conventional corrective or compensatory mechanics composed of brackets and wires.

## Supplementary Table 1

[Table t1app]

**Table t1app:** Terms selected to be used during searches, according to the P (participants) and E (exposure) of PECO strategy.

	Mesh terms	Entry terms		Free terms
P	“Orthodontic Appliances, Removable”	Removable Orthodontic Appliance Removable Orthodontic Appliances Clear Aligner Appliances Appliance, Clear Aligner Appliances, Clear Aligner Clear Aligner Appliance Invisalign -		-
	Orthodontic Appliances	Appliance, Orthodontic Appliances, Orthodontic Orthodontic Appliance		-
	Orthodontic Appliances, Fixed	Appliance, Fixed Orthodontic Appliances, Fixed Orthodontic Fixed Orthodontic Appliance Fixed Orthodontic Appliances Orthodontic Appliance, Fixed Fixed Functional Appliances Appliance, Fixed Functional Appliances, Fixed Functional	Fixed Functional Appliance Functional Appliance, Fixed Functional Appliances, Fixed Fixed Appliances Appliance, Fixed Appliances, Fixed Fixed Appliance	Braces Brackets
	Orthodontics	-		-
E	Mobile Applications	Application, Mobile Applications, Mobile Mobile Application Mobile Apps App, Mobile Apps, Mobile Mobile App Portable Electronic Apps App, Portable Electronic Apps, Portable Electronic Electronic App, Portable Electronic Apps, Portable Portable Electronic App Portable Electronic Applications Application, Portable Electronic Applications, Portable Electronic	Electronic Application, Portable Electronic Applications, Portable Portable Electronic Application Portable Software Apps App, Portable Software Apps, Portable Software Portable Software App Software App, Portable Software Apps, Portable Portable Software Applications Application, Portable Software Applications, Portable Software Portable Software Application Software Application, Portable Software Applications, Portable	Teledentistry Telemonitoring Distance monitoring Teleorthodontics Tele-orthodontics Home teleassistance Dental monitoring Remote monitoring Distance counseling Teleodontology Tele-odontology
	Smartphone	Smartphones Smart Phones Smart Phone Phones, Smart		-

## Supplementary Table 2

[Table t2app]

**Table t2app:** Searches performed in the databases of this systematic review.

Database	Search performed in the selected database
MEDLINE (Pubmed)	#1 (Orthodontic Appliances[MeSH Terms]) OR (Appliance, Orthodontic[Title/Abstract] OR Appliances, Orthodontic[Title/Abstract] OR Orthodontic Appliance[Title/Abstract]) OR Orthodontic Appliances, Removable[MeSH Terms] OR “Removable Orthodontic Appliance”[Title/Abstract] OR “Removable Orthodontic Appliances”[Title/Abstract] OR “Clear Aligner Appliances”[Title/Abstract] OR “Appliance, Clear Aligner”[Title/Abstract] OR “Appliances, Clear Aligner”[Title/Abstract] OR “Clear Aligner Appliance”[Title/Abstract] OR “Invisalign”[Title/Abstract] OR (Orthodontic Appliances, Fixed[MeSH Terms]) OR (Orthodontic Appliances, Fixed[Title/Abstract] OR Appliance, Fixed Orthodontic[Title/Abstract] OR Appliances, Fixed Orthodontic[Title/Abstract] OR Fixed Orthodontic Appliance[Title/Abstract] OR Fixed Orthodontic Appliances[Title/Abstract] OR Orthodontic Appliance, Fixed[Title/Abstract] OR Fixed Functional Appliances[Title/Abstract] OR Appliance, Fixed Functional[Title/Abstract] OR Appliances, Fixed Functional[Title/Abstract] OR Fixed Functional Appliance[Title/Abstract] OR Functional Appliance, Fixed[Title/Abstract] OR Functional Appliances, Fixed[Title/Abstract] OR Fixed Retainer[Title/Abstract] OR Fixed Retainers[Title/Abstract] OR Retainer, Fixed[Title/Abstract] OR Retainers, Fixed[Title/Abstract] OR Bonded Retainer[Title/Abstract] OR Bonded Retainers[Title/Abstract] OR Retainer, Bonded[Title/Abstract] OR Retainers, Bonded[Title/Abstract] OR Fixed Appliances[Title/Abstract] OR Appliance, Fixed[Title/Abstract] OR Appliances, Fixed[Title/Abstract] OR Fixed Appliance[Title/Abstract] OR Permanent Retainer[Title/Abstract] OR Permanent Retainers[Title/Abstract] OR Retainer, Permanent[Title/Abstract] OR Retainers, Permanent[Title/Abstract]) OR Orthodontics[MeSH Terms] #2 (Mobile Applications[MeSH Terms]) OR (Mobile Applications[Title/Abstract] OR Application, Mobile[Title/Abstract] OR Applications, Mobile[Title/Abstract] OR Mobile Application[Title/Abstract] OR Mobile Apps[Title/Abstract] OR App, Mobile[Title/Abstract] OR Apps, Mobile[Title/Abstract] OR Mobile App[Title/Abstract] OR Portable Electronic Apps[Title/Abstract] OR App, Portable Electronic[Title/Abstract] OR Apps, Portable Electronic[Title/Abstract] OR Electronic App, Portable[Title/Abstract] OR Electronic Apps, Portable[Title/Abstract] OR Portable Electronic App[Title/Abstract] OR Portable Electronic Applications[Title/Abstract] OR Application, Portable Electronic[Title/Abstract] OR Applications, Portable Electronic[Title/Abstract] OR Electronic Application, Portable[Title/Abstract] OR Electronic Applications, Portable[Title/Abstract] OR Portable Electronic Application[Title/Abstract] OR Portable Software Apps[Title/Abstract] OR App, Portable Software[Title/Abstract] OR Apps, Portable Software[Title/Abstract] OR Portable Software App[Title/Abstract] OR Software App, Portable[Title/Abstract] OR Software Apps, Portable[Title/Abstract] OR Portable Software Applications[Title/Abstract] OR Application, Portable Software[Title/Abstract] OR Applications, Portable Software[Title/Abstract] OR Portable Software Application[Title/Abstract] OR Software Application, Portable[Title/Abstract] OR Software Applications, Portable[Title/Abstract]) OR Distance counseling[Title/Abstract] OR Teledentistry[Title/Abstract] OR Telemonitoring[Title/Abstract] OR Distance monitoring[Title/Abstract] OR Teleorthodontics[Title/Abstract] OR Tele-orthodontics[Title/Abstract] OR home teleassistance[Title/Abstract] OR dental monitoring[Title/Abstract] OR remote monitoring[Title/Abstract] OR (Smartphone[MeSH Terms]) OR (Smartphone[Title/Abstract] OR Smartphones[Title/Abstract] OR Smart Phones[Title/Abstract] OR Smart Phone[Title/Abstract] OR Phones, Smart[Title/Abstract]) Final search: #1 AND #2
Scopus	( ( TITLE-ABS-KEY ( “Removable Orthodontic Appliance” OR “Removable Orthodontic Appliances” OR “Clear Aligner Appliance” OR “Appliance, Clear Aligner” OR “Appliances, Clear Aligner” OR “Clear Aligner Appliance” OR invisalign ) OR TITLE-ABS-KEY ( “Appliance, Orthodontic” OR “Appliances, Orthodontic” OR “Orthodontic Appliance” ) OR TITLE-ABS-KEY ( “Appliance, Fixed Orthodontic” OR “Appliances, Fixed Orthodontic” OR “Fixed Orthodontic Appliance” OR “Fixed Orthodontic Appliances” OR “Orthodontic Appliance, Fixed” OR “Fixed Functional Appliances” OR “Appliance, Fixed Functional” OR “Appliances, Fixed Functional” OR “Fixed Functional Appliance” OR “Functional Appliance, Fixed” OR “Functional Appliances, Fixed” OR “Fixed Retainer” OR “Fixed Retainers” OR “Retainer, Fixed” OR “Retainers, Fixed” OR “Bonded Retainer” OR “Bonded Retainers” OR “Retainer, Bonded” OR “Retainers, Bonded” OR “Fixed Appliances” OR “Appliance, Fixed” OR “Appliances, Fixed” OR “Fixed Appliance” OR “Permanent Retainer” OR “Permanent Retainers” OR “Retainer, Permanent” OR “Retainers, Permanent” ) OR TITLE-ABS-KEY ( “Orthodontics” ) ) ) AND ( ( TITLE-ABS-KEY ( “Application, Mobile” ) OR ( “Applications, Mobile” ) OR ( “Mobile Application” ) OR ( “Apps, Mobile” ) OR ( “Mobile App” ) OR ( “Portable Electronic Apps” ) OR ( “App, Portable Electronic” ) OR ( “Apps, Portable Electronic” ) OR ( “Electronic App, Portable” ) OR ( “Electronic Apps, Portable” ) OR ( “Portable Electronic App” ) OR ( “Portable Electronic Applications” ) OR ( “Application, Portable Electronic” ) OR ( “Applications, Portable Electronic” ) OR ( “Electronic Application, Portable” ) OR ( “Electronic Applications, Portable” ) OR ( “Portable Electronic Application” ) OR ( “Portable Software Apps” ) OR ( “App, Portable Software” ) OR ( “Apps, Portable Software” ) OR ( “Portable Software App” ) OR ( “Software App, Portable” ) OR ( “Software Apps, Portable” ) OR ( “Portable Software Applications” ) OR ( “Application, Portable Software” ) OR ( “Applications, Portable Software” ) OR ( “Portable Software Application” ) OR ( “Software Application, Portable” ) OR ( “Software Applications, Portable” ) ) OR TITLE-ABS-KEY ( ( “Distance counseling” ) OR ( “Teledentistry” ) OR ( “Telemonitoring” ) OR ( “Distance monitoring” ) OR ( “Teleorthodontics” ) OR ( “Tele-orthodontics” ) OR ( “home teleassistance” ) OR ( “dental monitoring” ) OR ( “remote monitoring” ) ) OR TITLE-ABS-KEY ( ( “Smartphone” ) OR ( “Smartphones” ) OR ( “Smart Phones” ) OR ( “Smart Phone” ) OR ( “Phones, Smart” ) ) )
Web of Science	#1 “Removable Orthodontic Appliance” OR “Removable Orthodontic Appliances” OR “Clear Aligner Appliance*” OR “Appliance, Clear Aligner” OR “Appliances, Clear Aligner” OR “Clear Aligner Appliance” OR Invisalign (Topic) or “Appliance, Orthodontic” OR “Appliances, Orthodontic” OR “Orthodontic Appliance*” (Topic) or “Appliance, Fixed Orthodontic*” OR “Appliances, Fixed Orthodontic*” OR “Fixed Orthodontic Appliance*” OR “Fixed Orthodontic Appliances” OR “Orthodontic Appliance, Fixed” OR “Fixed Functional Appliances” OR “Appliance, Fixed Functional” OR “Appliances, Fixed Functional” OR “Fixed Functional Appliance” OR “Functional Appliance, Fixed” OR “Functional Appliances, Fixed” OR “Fixed Retainer” OR “Fixed Retainers” OR “Retainer, Fixed” OR “Retainers, Fixed” OR “Bonded Retainer” OR “Bonded Retainers” OR “Retainer, Bonded” OR “Retainers, Bonded” OR “Fixed Appliances” OR “Appliance, Fixed” OR “Appliances, Fixed” OR “Fixed Appliance” OR “Permanent Retainer” OR “Permanent Retainers” OR “Retainer, Permanent” OR “Retainers, Permanent” (Topic) or Orthodontic* (Topic) #2 “Application, Mobile” OR “Applications, Mobile” OR “Mobile Application” OR “Apps, Mobile” OR “Mobile App” OR “Portable Electronic Apps” OR “App, Portable Electronic” OR “Apps, Portable Electronic” OR “Electronic App, Portable” OR “Electronic Apps, Portable” OR “Portable Electronic App” OR “Portable Electronic Applications” OR “Application, Portable Electronic” OR “Applications, Portable Electronic” OR “Electronic Application, Portable” OR “Electronic Applications, Portable” OR “Portable Electronic Application” OR “Portable Software Apps” OR “App, Portable Software” OR “Apps, Portable Software” OR “Portable Software App” OR “Software App, Portable” OR “Software Apps, Portable” OR “Portable Software Applications” OR “Application, Portable Software” OR “Applications, Portable Software” OR “Portable Software Application” OR “Software Application, Portable” OR “Software Applications, Portable” (Topic) or “Distance counseling” OR Teledentistry OR Telemonitoring OR “Distance monitoring” OR Teleorthodontics OR Tele-orthodontics OR “home teleassistance” OR “dental monitoring” OR “remote monitoring” (Topic) or Smartphone OR Smartphones OR “Smart Phones” OR “Smart Phone” OR “Phones, Smart” (Topic) Final search #1 AND #2
The Cochrane Library	ID Search #1 (Appliance, Removable Orthodontic OR Removable Orthodontic Appliance OR Removable Orthodontic Appliances OR Clear Aligner Appliances OR Appliance, Clear Aligner OR Appliances, Clear Aligner OR Clear Aligner Appliance OR Invisalign):ti,ab,kw #2 (Appliance, Orthodontic OR Appliances, Orthodontic OR Orthodontic Appliance):ti,ab,kw #3 (Appliance, Fixed Orthodontic OR Appliances, Fixed Orthodontic OR Fixed Orthodontic Appliance OR Fixed Orthodontic Appliances OR Orthodontic Appliance, Fixed OR Fixed Functional Appliances OR Appliance, Fixed Functional OR Appliances, Fixed Functional OR Fixed Functional Appliance OR Functional Appliance, Fixed OR Functional Appliances, Fixed OR Fixed Retainer OR Fixed Retainers OR Retainer, Fixed OR Retainers, Fixed OR Bonded Retainer OR Bonded Retainers OR Retainer, Bonded OR Retainers, Bonded OR Fixed Appliances OR Appliance, Fixed OR Appliances, Fixed OR Fixed Appliance OR Permanent Retainer OR Permanent Retainers OR Retainer, Permanent OR Retainers, Permanent):ti,ab,kw #4 #1 OR #2 OR #3 #5 (Application, Mobile OR Applications, Mobile OR Mobile Application OR Mobile Apps OR App, Mobile OR Apps, Mobile OR Mobile App OR Portable Electronic Apps OR App, Portable Electronic OR Apps, Portable Electronic OR Electronic App, Portable OR Electronic Apps, Portable OR Portable Electronic App OR Portable Electronic Applications OR Application, Portable Electronic OR Applications, Portable Electronic OR Electronic Application, Portable OR Electronic Applications, Portable OR Portable Electronic Application OR Portable Software Apps OR App, Portable Software OR Apps, Portable Software OR Portable Software App OR Software App, Portable OR Software Apps, Portable OR Portable Software Applications OR Application, Portable Software OR Applications, Portable Software OR Portable Software Application OR Software Application, Portable OR Software Applications, Portable):ti,ab,kw #6 (Distance counseling OR Teledentistry OR Telemonitoring OR Distance monitoring OR Teleorthodontics OR Tele-orthodontics OR home teleassistance OR dental monitoring OR remote monitoring):ti,ab,kw #7 (Smartphone OR Smartphones OR Smart Phones OR Smart Phone OR Phones, Smart):ti,ab,kw #8 #5 OR #6 OR #7 #9 #4 AND #8
Virtual Health Library (VHL)	((Removable Orthodontic Appliance) OR (Removable Orthodontic Appliances) OR (Clear Aligner Appliances) OR (Appliance, Clear Aligner) OR (Appliances, Clear Aligner) OR (Clear Aligner Appliance) OR Invisalign) OR ((Appliance, Orthodontic) OR (Appliances, Orthodontic) OR (Orthodontic Appliance)) OR (Orthodontics) OR ((Appliance, Fixed Orthodontic) OR (Appliances, Fixed Orthodontic) OR (Fixed Orthodontic Appliance) OR (Fixed Orthodontic Appliances) OR (Orthodontic Appliance, Fixed) OR (Fixed Functional Appliances) OR (Appliance, Fixed Functional) OR (Appliances, Fixed Functional) OR (Fixed Functional Appliance) OR (Functional Appliance, Fixed) OR (Functional Appliances, Fixed) OR (Fixed Retainer) OR (Fixed Retainers) OR (Retainer, Fixed) OR (Retainers, Fixed) OR (Bonded Retainer) OR (Bonded Retainers) OR (Retainer, Bonded) OR (Retainers, Bonded) OR (Fixed Appliances) OR (Appliance, Fixed) OR (Appliances, Fixed) OR (Fixed Appliance) OR (Permanent Retainer) OR (Permanent Retainers) OR (Retainer, Permanent) OR (Retainers, Permanent))) AND (((Application, Mobile) OR (Applications, Mobile) OR (Mobile Application) OR (Apps, Mobile) OR (Mobile App) OR (Portable Electronic Apps) OR (App, Portable Electronic) OR (Apps, Portable Electronic) OR (Electronic App, Portable) OR (Electronic Apps, Portable) OR (Portable Electronic App) OR (Portable Electronic Applications) OR (Application, Portable Electronic) OR (Applications, Portable Electronic) OR (Electronic Application, Portable) OR (Electronic Applications, Portable) OR (Portable Electronic Application) OR (Portable Software Apps) OR (App, Portable Software) OR (Apps, Portable Software) OR (Portable Software App) OR (Software App, Portable) OR (Software Apps, Portable) OR (Portable Software Applications) OR (Application, Portable Software) OR (Applications, Portable Software) OR (Portable Software Application) OR (Software Application, Portable) OR (Software Applications, Portable)) OR ((Distance counseling) OR Teledentistry OR Telemonitoring OR (Distance monitoring) OR Teleorthodontics OR Tele-orthodontics OR (home teleassistance) OR (dental monitoring) OR (remote monitoring)) OR (Smartphone OR Smartphones OR (Smart Phones) OR (Smart Phone) OR (Phones, Smart)))
Google Scholar	Any idiom; Without patents and citations; Classified by relevance; Search; “orthodontics + teledentistry”
OpenGrey	Tele-orthodontics
